# Self-organizing map models of language acquisition

**DOI:** 10.3389/fpsyg.2013.00828

**Published:** 2013-11-19

**Authors:** Ping Li, Xiaowei Zhao

**Affiliations:** ^1^Department of Psychology and Center for Brain, Behavior, and Cognition, Pennsylvania State University, University ParkPA, USA; ^2^Department of Psychology, Emmanuel CollegeBoston, MA, USA

**Keywords:** SOM, connectionism, language acquisition, vocabulary spurt, lexical aspect, age of acquisition, cross-language priming

## Abstract

Connectionist models have had a profound impact on theories of language. While most early models were inspired by the classic parallel distributed processing architecture, recent models of language have explored various other types of models, including self-organizing models for language acquisition. In this paper, we aim at providing a review of the latter type of models, and highlight a number of simulation experiments that we have conducted based on these models. We show that self-organizing connectionist models can provide significant insights into long-standing debates in both monolingual and bilingual language development. We suggest future directions in which these models can be extended, to better connect with behavioral and neural data, and to make clear predictions in testing relevant psycholinguistic theories.

## INTRODUCTION

The parallel distributed processing (PDP) models have stimulated tremendous interests in computational models of language and led to intense debates regarding the nature and representation of language. Today, more than a quarter century after the original PDP volumes (McClelland et al., 1986; Rumelhart and McClelland, 1986), connectionism has become a powerful tool as well as a conceptual framework for us to understand many important issues in language learning, processing, and impairment. According to the connectionist framework, many critical aspects of human cognition are emergent properties, and language is an example *par excellence*. Language as a hallmark of human behavior thus received in-depth treatment in the original PDP volumes, and connectionist language models have flourished in the last 25 years. It is important to note that these models may involve significantly different computational architectures, for example, with regard to both representation structures (e.g., localist vs. distributed representation) or learning mechanisms (supervised vs. unsupervised learning). In this article, we focus on a type of unsupervised connectionist learning models, the self-organizing maps (SOMs)^3^, and illustrate ways in which SOM-based connectionist models can be used effectively to study the acquisition and processing of both first and second languages.

### SELF-ORGANIZING MAPS

In contrast to the classic PDP learning models (e.g., of the type learned via back-propagation), unsupervised learning models use no explicit error signals to adjust the weights between input and output. These models span a wide range of learning algorithms, including the Adaptive Resonance Theory (ART; Grossberg, 1976a,b; see Hinton and Sejnowski, 1999 for a collection of unsupervised models). Here we focus on a particular unsupervised learning algorithm called SOM (Kohonen, 2001), which has been widely used in modeling language learning and representation (see Li and Zhao, 2012 for a bibliography).

**FIGURE 1:**
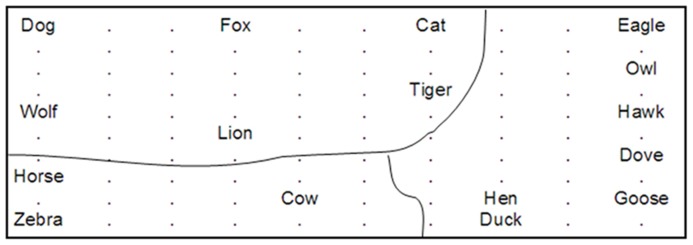
**An illustration of the learned semantic categories in a SOM model.** Concepts with similar features/attributes are grouped together such as *horse* and *zebra*.

A standard SOM consists of a two-dimensional topographic map for the organization of input representations, where each node is a unit on the map that receives input via the input-to-map connections. At each training step of SOM, an input pattern (e.g., the phonological or semantic information of a word) is randomly picked out and presented to the network, which activates many units on the map, initially randomly. The SOM algorithm starts out by identifying all the incoming connection weights to each and every unit on the map, and for each unit, compares the weight vector with the input vector. If the unit’s weight vector and the input vector are similar or identical by chance, the unit will receive the highest activation and is declared the “winner” (the Best Matching Unit or BMU, see **Figure 1** for an example). Once a unit becomes highly active for a given input, its weight vector and that of its neighboring units are adjusted, such that they become more similar to the input and hence will respond to the same or similar inputs more strongly the next time. In this way, every time an input is presented, an area of nodes will become activated on the map (the “activity bubbles”) and the maximally active nodes are taken to represent the input.

Equation 1 shows how the activations of the nodes on the map are calculated. Considering a node *k* that has a vector *m*_k_ associated with it to represent the weights of the input connections to it. Given an input vector **x** (e.g., the phonological or semantic information of a word), the localized output response α of node *k* is computed as:

(1) $$\alpha_{k}=\left\{ \begin{array}{l} 1-\frac{∥x-m_{k}∥-d_{\min}}{d_{\max}-d_{\min}},\;if\;k\in N_{c}\\ \;\;\;\;\;0,\;\;\;\;\;\text{otherwise} \end{array}\right.,\;\;\;$$

where *N*_c_ is the set of neighbors of the winner *c* [for which α**_c_ = max**_k_(α**_k_) = 1], *d*_ min_ and *d*_ max_ are the smallest and the largest Euclidean distances of **x** to node’s weight vectors within *N*_c_. Initially activation occurs in large areas of the map, that is, large neighborhoods, but gradually learning becomes focused and the size of the neighborhoods reduces to only one node (the winner), which has an activation level of one. This process continues until all the input patterns elicit specific response units in the map (i.e., the BMUs).

As a result of this self-organizing process, the statistical structure implicit in the input is captured by the topographic structure of the SOM. In this newly formed topographic structure (the new representation), similar inputs will end up activating nodes in nearby regions, yielding meaningful activity bubbles that can be visualized on the 2-D space of the map. Equation 2 shows how the weights of the nodes around a winner or BMU are updated:

(2) $$m_{k}\,\left(t+1\right)=m_{k}\,\left(t\right)+\beta \,\left(t\right)\cdot \left[x-m_{k}\,\left(t\right)\right]\,\;f\,o\,r\,\;a\,l\,l\,\;k\in N_{c}\,.$$

Here, β(*t*) is the learning rate for the map, which changes with time *t*. If the node *k* belongs to the nodes in the neighborhood of the winner *c*, its weight should be adjusted according to this equation; otherwise, it remains unchanged.

Self-organizing maps have several important properties that make them particularly well suited to the study of language acquisition. First, as unsupervised learning systems, SOMs require no explicit teacher; learning is achieved by the system’s organization in response to the input. Such networks provide computationally relevant models for language acquisition, given that in real language learning children do not receive constant feedback about what is incorrect in their speech (such as the kind of error corrections provided by supervised learning algorithms). Second, self-organization in these networks allow for the gradual formation of structures as changes of activity bubbles on 2-D maps, as a result of extracting an efficient representation of the complex statistical regularities inherent in the high-dimensional input space (Kohonen, 2001). In particular, the network organizes information first in large areas of the map and gradually zeros in on to smaller areas (decreasing neighborhoods); this zeroing-in is a process from diffuse to focused patterns, as a function of the network’s continuous adaptation to the input characteristics. Third, the SOM can fall into a topography-preserving state once learning is achieved, which means nearby areas in the map respond to inputs with similar features. This property is consistent with known topographic features of certain areas of the brain where topographic maps are formed, especially in the primary motor, visual, and somatosensory cortical areas (Haykin, 1999; Spitzer, 1999; Miikkulainen et al., 2005). Although the association cortex in the human brain may be much more dynamic and less topographically organized (see Sporns, 2010), the SOM architecture does allow researchers to model the emergence of higher-level cognitive processes (see Miikkulainen, 1993, 1997), including the emergence of lexical categories as a gradual process and natural outcome of language learning (see Li, 2003).

As in other computational models, training in a SOM involves the use and manipulation of free parameters such as map size (number of nodes in the network), neighborhood size (initial radius of the training nodes), learning rate, etc. Appropriate values of these parameters often lead to fast convergence of training or better overall performance of the model, and decisions need to be made by the modeler in advance, given the nature and complexity of the modeling task. As a general yardstick, for example, the size of the map should generally be three to four times the size of the input units to be learned, whereas the size of the initial radius should be sufficiently large (e.g., 1/4 of the map size) to allow for reorganization of the map’s topography, depending on how much plasticity the modeler wants to give to the network^4^. Both neighborhood size and learning rate in most SOM models take a linear decrease function (e.g., Miikkulainen, 1997), although some studies have tied the change of neighborhood size to quantization errors of the network (see Li et al., 2007 and discussion below). The modeler must constantly evaluate the impact of different values of the free parameters in affecting the performance of the model and speed of convergence. As an example, Richardson and Thomas (2008) systematically examined the influence of the free parameters, along with some other factors, on SOM’s ability to simulate critical periods in cognitive development.

### HEBBIAN LEARNING RULE

A highly influential learning mechanism that has also been computationally implemented in connectionist models is the Hebbian learning rule, due to the Canadian neuroscientist Donald Hebb (Hebb, 1949). In considering how biological neural networks could work, Hebb hypothesized that when neuron A is persistently and repeatedly engaged in exciting neuron B, the efficiency of A in firing B will be increased due to some growth process or metabolic changes taking place in one or both neurons. In other words, the strength between A and B is increased as a result of their frequent associations in neural activities. Hebb’s hypothesis is the basis of the slogan “neurons that fire together wire together.” It provides an important background for connectionism, as well as a biologically plausible mechanism for how associative learning and memory could occur at the neural level, as it is clearly related to long-term potentiation (LTP) in biological systems (Munakata and Pfaffly, 2004). Mathematically, the Hebbian learning rule can be expressed as Eq. 3:

(3) $$\Delta \,w_{k\,l}=\beta \,.\,\alpha_{k}\,.\,\alpha_{l},$$

where β is a constant learning rate, and Δ*w*_kl_ refers to change of weights from input *k* to *l* and α**_k_ and α**_l_ the associated activations of neurons *k* and *l*. The equation indicates that the connection strengths between neurons *k* and *l* will be increased as a function of their concurrent activities.

Although the Hebbian learning rule itself is not explicitly included in the SOM algorithm discussed above, it has been a very useful mechanism for connectionist language models based on SOM. In particular, several SOMs can be linked together via associative connections trained by Hebbian learning (see Miikkulainen, 1993, 1997 for this approach). As shown in several models discussed next, when Hebbian learning is incorporated, the SOM model has strong implications for language acquisition: it can account for the process of how the learner establishes relationships between word forms, lexical semantics, and grammatical morphology, on the basis of how often they co-occur and how strongly they are co-activated in the representation.

## SOM-BASED CONNECTIONIST MODELS OF LANGUAGE

### MODELS WITH SINGLE SOMs

Many early SOM-based connectionist models include just one layer of SOM, which usually only accounts for a particular aspect of language in which the researchers are interested. A good example is the classic work of Ritter and Kohonen (1989) that demonstrates that SOM networks can capture the semantic structure of words. These authors tested a single SOM with inputs representing the meaning of words that were generated from two methods. The first method is a feature-based method, according to which a word’s meaning is represented by a vector and each dimension of this vector represents a possible descriptive feature or attribute of the concept. The value of the dimension could be 0 or 1, indicating the absence (0) or presence (1) of a particular feature for the target word. For example, the representations of *dove* and *hen* are very similar except one dimension representing the flying feature (dove = 1, hen = 0). Specifically, Ritter and Kohonen (1989) generated a detailed representation of 16 animals based on 13 attributes, trained a SOM with the 16 animal words, and found that the network was able to form topographically organized representations of semantic categories associated with the 16 animal words; see an example in **Figure 1**.

Ritter and Kohonen’s (1989) second method of representing meanings of words is a statistics-based method, according to which the researchers generated a corpus consisting of three-word sentences randomly formed from a list of nouns, verbs, and adverbs (e.g., *Dog drinks fast*). A trigram window is applied to the corpus, and the co-occurrence frequencies of the word in the middle of the trigram with its two closest neighbors are calculated. This generates a co-occurrence matrix, which forms the basis of each word’s “average context vector,” a combination of the average of all the words preceding the target word and that of all the words following it. The researchers then used these vectors as input to the SOM, and training on the SOM again indicated topographically structured semantic/grammatical categories on the map, like the example shown in **Figure 1**. Ritter and Kohonen’s (1989) pioneering work clearly shows that categories implicitly in the linguistic environment (input streams) can be extracted by the SOM^5^. The properties of a topographic-preserving map as demonstrated by Ritter and Kohonen (1989) provide the basis for SOM as a model to simulate empirical findings regarding semantic representation and semantic priming (see Spitzer, 1999 and discussions of SEMANT and DevLex-II below).

Silberman et al. (2007) introduced the SEMANT model to simulate the associations of words/concepts in a semantic network. SEMANT includes one SOM that handles semantic information that was extracted from large-scale corpora based on the method of Li et al. (2000) using the CHILDES database (MacWhinney, 2000). SEMANT also integrates a component of episodic memory simulated by the lateral connections among the units on the SOM. The basic idea here was that the semantically related words tend to occur together in a linguistic content, and therefore their episodic associations tend to be strong. Simulation results for the model showed that SEMANT was able to replicate the empirical findings from psycholinguistics, such as effects of semantic priming that indicate faster response to the related word than unrelated words (e.g., faster lexical decision times for *nurse* than to *bread* upon hearing *doctor*; Neely and Durgunoglu, 1985).

In addition to semantic learning, SOM networks have also been used for simulating phonological development. For example, Guenther and Gjaja (1996) introduced a single-layer SOM to simulate the “perceptual magnet effect” (Kuhl, 1991) in infants’ phonetic learning. In particular, the authors first trained the SOM (or “auditory map” as so named by the authors) with input sound patterns which contained formant information of different phonemic categories (such as /r/ and /l/ in American English). They then presented the network with test sounds similar to the phonemes that the network was trained on. When a test stimulus was presented to the map, the activities of all nodes on the map were calculated. Each node’s activity level was further used to multiply its “preferred stimulus” (the input vector that activated the node most strongly), and the resulting products for all the nodes were added together and normalized to serve as the map’s “population vector.” Guenther and Gjaja (1996) measured the population vectors that corresponded to each test stimulus, and used these measures to represent the map’s overall perception of the particular test stimulus. Consistent with results from empirical studies of human listeners, the SOM-based modeling results showed a warpping of perceptual space, that is, the acoustic patterns near the center/prototype of the learned sound categories are perceived as more similar to each other than to those patterns further away from the center, a trademark of “perceptual magnet effect.”

### MODELS WITH MULTIPLE SOMs

Although connectionist models with only one layer of SOM have be successful in simulating different aspects of language one at a time (e.g., semantic learning or phonological learning), researchers have realized that in natural language contexts the user or learner is engaged in a process in which phonological, lexical, semantic, and orthographic information often occurs simultaneously. An integrated SOM-based model must be able to simulate this process. Multiple SOMs that are interconnected have thus been developed in response to this requirement.

One of the earliest attempts to construct a full-scale multiple SOM language model was Miikkulainen (1997; see also Miikkulainen, 1993). He introduced the DISLEX model, which includes different SOMs connected through associative links via Hebbian learning. In DISLEX, each map is dedicated to a specific type of linguistic information (e.g., orthography, phonology, and semantics), and is trained as a standard SOM. In the training of the network, an input pattern activates a node or a group of nodes on one of the input maps, and the resulting activity bubble propagates through the associative links and causes an activity bubble to form in the other map. The activation of co-occurring lexical and semantic representations leads to continuous organization in these maps, and most importantly, to adaptive formations of associative connections between the maps. The DISLEX model was successfully used to simulate certain impaired language processes such as dyslexia and aphasia (Miikkulainen, 1997), and has also been applied to simulate bilingual representation (Miikkulainen and Kiran, 2009), bilingual aphasia (Kiran et al., 2013), and the acquisition of Chinese reading by elementary school children (Xing et al., 2004).

Using the basic idea of multiple SOMs connected via associative links, Li et al. (2004) developed the Developmental Lexicon (DevLex) model to simulate children’s early lexical development. Similar to DISLEX, DevLex is a multi-layer self-organizing model with cross-layer links trained by Hebbian learning. Unlike DISLEX that uses the standard SOM learning algorithm, it includes two growing SOMs which recruit additional nodes in response to task demands in learning, and these new nodes are inserted in the topographic structure of the existing map as the network’s learning progresses. The growth of new nodes is dependent upon accuracy of learning (e.g., as more errors occur more nodes are inserted). One growing maps handles the semantic representation and another the phonological representation of words. DevLex takes advantages of the SOM properties discussed earlier (see Introduction). The Li et al. (2004) simulations showed that it developed topographically organized representations for linguistic categories over time, modeled lexical confusion as a function of word density and semantic similarity, and displayed age-of-acquisition effects in the course of learning a growing lexicon. These results matched up with patterns from empirical studies of children’s early lexical development. DevLex later evolved into the DevLex-II model, which we will discuss in the next section (Li et al., 2007).

Mayor and Plunkett (2010) introduced a self-organizing model to account for fast mapping in early word learning. Their simulations particularly focused on the generalization property of word–object associations based on the taxonomic/categorical relationship of objects. Their model included two SOMs with one receiving visual input (the *object*) and another acoustic input (the *word*). The connections of the two maps were adjusted by Hebbian learning rule, which emphasizes that the cross-layer weights are reinforced as the object and the word are simultaneously presented to their model. Mayor and Plunkett (2010) pointed out that this joint presentation of an object and its corresponding name reflected the results of the development of infants’ joint-attentional activities with their caregivers. Although the visual inputs to this model were random dot matrices artificially generated, the model could simulate several interesting patterns in children’s early lexical and category development, such as the taxonomic constraint that indicates children tend to give a new object a known name in the same category (e.g., seeing a tiger for the first time and immediately call it a cat, which they already learned). The authors also argued that an efficient, pre-established categorization capacity is a prerequisite to successful word learning. This argument is highly consistent with data from other simulations of early lexical development such as those based on DevLex-II (see discussion under the Section “Modeling Vocabulary Spurt”).

Recently, Kiran et al. (2013) presented data from the DISLEX model that simulated patterns of bilingual language recovery in aphasic patients. A distinct feature of the Kiran et al.’s (2013) model was that they applied it to simulate empirical patterns of, on a case-by-case basis, each of the 17 patients who underwent treatment following injury. Their simulation results showed a close match with real behavioral data from individual patients, and this is a testimony that computational models can closely reflect realistic linguistic processes from realistic language users (rather than from simplified or idealized situations). In order to do so, Kiran et al.’s (2013) model incorporated important variables underlying patterns of behavior, including the patient’s language history with regard to age of L1 and L2 acquisition, proficiency, and the dominance of the treatment language. More impressive was the model’s ability to predict the efficacy of rehabilitation in each of the bilingual’s languages. In reality, each bilingual patient underwent rehabilitation treatment for only one of their languages (English or Spanish) due to empirical constraints, but the computational model was trained for recovery in both languages following lesion, thus showing considerable advantage and flexibility of the model as compared with examination of the actual patient. It is important to note that in empirical studies the researcher, when faced with the injured patient, cannot go back to study the patient’s pre-lesion condition, whereas in computational modeling the researcher can examine the intact model, lesion it, and track the performance of the same model before and after lesion, as was done by Kiran et al. (2013) in their study.

## DevLex-II: A SCALABLE SOM-BASED CONNECTIONIST MODEL OF LANGUAGE

In this section, we present the details of the DevLex-II model (Li et al., 2007), a SOM-based connectionist model designed to simulate processes of language learning in both the monolingual and bilingual situations. In a number of studies (Zhao and Li, 2009, 2010, 2013), we have tested the model’s ability in accounting for patterns of first (L1) and second (L2) language acquisition. We say that the model is “scalable” because it can be used to simulate a large realistic linguistic lexicon, in single or multiple languages, and for various bilingual language pairs (such as Chinese–English, Spanish–English, etc.). In what follows we will first discuss some key features of the DevLex-II architecture and then discuss the applications of the model to various L1 and L2 phenomena to illustrate how models based on multiple SOMs can be used effectively to address critical issues in L1 and L2 acquisition.

### ARCHITECTURE OF THE MODEL

**FIGURE 2:**
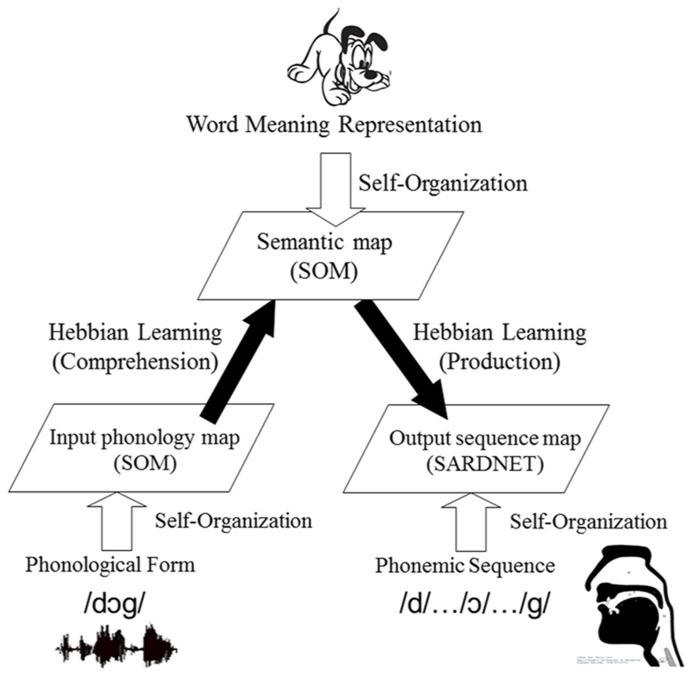
**A sketch of the DevLex-II model.** See text for explanation of the architecture of the model.

Considering the features of previous models (DISLEX, DevLex), the DevLex-II model builds on the basic structure as described above: multiple SOMs which are connected via Hebbian learning. The architecture of the model is illustrated in **Figure 2**. The model includes three basic levels for the representation and organization of linguistic information: phonological content, semantic content, and the articulatory output sequence of the lexicon. The core of the model is a SOM that handles lexical-semantic representation. This SOM is connected to two other SOMs, one for input (auditory) phonology, and another for articulatory sequences of output phonology. Upon training of the network, the semantic representation, input phonology, and output phonemic sequence of a word are simultaneously presented to the network. This process can be analogous to that of a child hearing a word and performing analysis of its semantic, phonological, and phonemic information.

On the semantic and phonological levels, the network constructs the representations based on the corresponding linguistic input according to the standard SOM algorithm. On the phonemic output level, DevLex-II uses an algorithm called SARDNET (James and Miikkulainen, 1995), a SOM-based temporal sequence learning network. The addition of the SARDNET algorithm to the model is based on considerations that word production is a temporal sequence ordering problem, and that language learners face the challenge to develop better articulatory control of the phonemic sequences of words.

In this architecture, at each training step, phonemes are input into the sequence map one by one, according their order of occurrence in a word. The winning unit of a phoneme is found and the weights of nodes in its neighborhood are adjusted; meanwhile, the activation levels of the winners responding to phonemes preceding the current phoneme will be adjusted by a number γ**^d^, where γ is a constant and *d* is the distance between the location of the current phoneme and the preceding phoneme that occurred in the word. This adjustment is intended to model the effect of phonological short-term memory during the learning of articulatory sequences; the activation of the current phoneme could be accompanied by some rehearsal of previous phonemes due to phonological short-term memory, which deepens the network’s or the learner’s knowledge of previous phonemes. The γ here is chosen to be <1 (0.8 in our case), in order to model the fact that phonological short-term memory tends to decay with time. For a word with *n* phonemes, the output of the winner responding to the *j*^ th^ phoneme will be 1 + γ + γ^2^ +……+ γ^*n-j*^, which is a geometric progression, and can be written as:

(4) $$\alpha_{w\,i\,n\,n\,e\,r}\,\left(j\right)=\frac{\left(1-\gamma^{n-j+1}\right)}{1-\gamma }\,.$$

According to this equation, when the representation of all the phonemes in a word is received by the output sequence map, the activation of some nodes (e.g., the first winner) will be larger than 1, so they need to be normalized to the range between 0 and 1. Thus, the node in response to the first phoneme of the word will have the largest activation, followed by sequentially decaying activations of other phonemes in the sequence of the word.

In DevLex-II, the associative connections between maps are trained via the Hebbian learning rule, as in DevLex and DISLEX. As training progresses, the weights of the associative connections between the frequently and concurrently activated nodes on two maps will become increasingly stronger. After the cross-map connections are stabilized, the activation of a word form can evoke the activation of a word meaning via form-to-meaning links, which models word comprehension. If the activated unit on the semantic map matches the correct word meaning, we determine that the network correctly comprehends this word; otherwise the network makes a comprehension error. Similarly, the activation of a word meaning can trigger the activation of an output sequence via meaning-to-sequence links, which models word production. If the activated units on the phonemic map match the phonemes making up the word in the correct order, we determine that the network correctly produces this word; otherwise the network makes a production error.

### PLASTICITY AND STABILITY IN THE MODEL

Since we aim at designing a scalable model that is suitable to simulate learning in different linguistic contexts (monolingual and bilingual), we must consider a fundamental problem called “catastrophic interference” (see French, 1999 for a review). Keeping the network’s plasticity for learning new words often causes it to lose its stability for old knowledge; conversely, a network that is too stable often cannot adapt itself very well to the new learning task. This problem has been termed the “plasticity–stability” dilemma in neural networks since the 1970s (Grossberg, 1976a,b). To resolve this problem for our studies (in particular the bilingual learning studies discussed below), we introduced two new features into DevLex-II.

The first is a self-adjustable *neighborhood function*. In the standard algorithm of SOM, the radius of the neighborhood usually decreases according to a fixed training timetable (see earlier discussion on SOM modeling parameters). This type of development in the network, though practically useful, is subject to the criticisms that (1) learning is tied directly and only to time (amount) of training, but is independent of the input-driven self-organizing process; and (2) the network often loses its plasticity for learning new inputs when the neighborhood radius becomes very small. DevLex-II considered these potential problems by using a learning process in which the neighborhood size is not totally locked with time, but is adjusted according to the network’s learning outcome (experience). In particular, neighborhood function depends on the network’s error level on each layer averaged across all the input patterns. Here, a “quantization error” (as used in Kohonen, 2001) of an input pattern is defined as the Euclidean distances of the input pattern to the weight vector of its winner or BMU.

A second way in which we have attempted to solve the plasticity–stability problem is to manage the training process to be more realistic for learning: for the input phonology map and the semantic map, during each training epoch, once a unit is activated as a BMU, it will become ineligible to respond to other inputs in the current training epoch. In this way, the old words are kept untouched in the training, whereas the new words can be represented by new units on the maps. A similar procedure is also used for the output sequence map at the word level, where the same phoneme in different locations of a word will be mapped to different, but adjacent, nodes on the map. This mechanism resembles DevLex’s growing map process in which new nodes are recruited for novel inputs as computational resources become scarce (see Li et al., 2004 for an algorithm in new node recruitment, as also discussed earlier). The use of a different but adjacent unit for new input is also empirically plausible: psycholinguistic research suggests that when young children encounter a novel word they tend to map it to a different category or meaning for which the child has not yet acquired a name (Markman, 1994; Mervis and Bertrand, 1994).

### LINGUISTIC REALISM OF THE MODEL

Many connectionist models of language are based on the use of artificially generated lexicon that is often limited in size. Such use of synthetic or highly simplified vocabularies provides certain modeling conveniences, but it lacks linguistic realism and is out of touch with the learner’s true lexical experience. As a step forward, we considered two methods in which our modeling data was constructed. First, in all of our studies with DevLex-II (Li et al., 2007; Zhao and Li, 2009, 2010, 2013), we used input based on realistic linguistic stimuli. For example, in several studies our simulation material was based on the vocabulary from the MacArthur–Bates Communicative Development Inventories (the CDI; Dale and Fenson, 1996), which allowed us to model a lexicon size of up to 1000 words. Second, we coded the input to our model as vector representation of the phonemic, phonological, or semantic information of words, extracted in the following ways: (1) PatPho, a generic phonological representation system, was used to generate the sound patterns of words based on articulatory features of different languages (Li and MacWhinney, 2002; Zhao and Li, 2010); (2) statistics-based methods were used to generate semantic representations of training stimuli from large-scale corpus data (e.g., CHILDES database; MacWhinney, 2000) or from computational thesauruses (e.g., WordNet database; Miller, 1990), as done in Li et al. (2007) and Zhao and Li (2009, 2010, 2013; see also earlier discussion about generating semantic representations in SOM-based models). Given the input representations constructed in the above manner, the DevLex-II model receives each representation sequentially in the training (i.e., one word at a time, in randomized order of training), approximating the word learning process in the realistic learning environment.

### DevLex-II MODELS OF MONOLINGUAL LANGUAGE ACQUISITION

Many interesting empirical phenomena have been examined in the field of monolingual language development; for example, in the study of lexical development, researchers have investigated patterns such as the vocabulary spurt, age of acquisition (AoA) of vocabulary, relationship between comprehension and production, motherese and role of input, word frequency effect, lexical category development, fast mapping, lexical overextension, U-shaped development, and so on (see Clark, 2009; Saxton, 2010). Connectionist approaches have been fruitfully applied to study these phenomena in the past two decades (see Westermann et al., 2009 for a review; see Li and Zhao, 2012 for a bibliography). The DevLex-II model was originally designed to account for several of the phenomena listed above.

#### Modeling vocabulary spurt

Vocabulary spurt refers to a period of extremely rapid growth of vocabulary starting around 18–24 months of age in children. A large number of studies have examined vocabulary spurt in young children (see Goldfield and Reznick, 1990; Bates and Carnevale, 1993 for example). Despite the empirical research in documenting the outcome of timing of vocabulary spurt, the underlying mechanisms for when and how vocabulary spurt occurs has been an issue of intense debate. To provide a computational account of this phenomenon, Li et al. (2007) trained a DevLex-II model to learn 591 English words extracted from the toddler’s vocabulary list of the English CDI. Their model incorporated several key features of learning and representation for lexical development, including the multiple SOMs that were used for simulating comprehension and production for the same items, along with realistic phonological and semantic input patterns of the lexical items.

**FIGURE 3:**
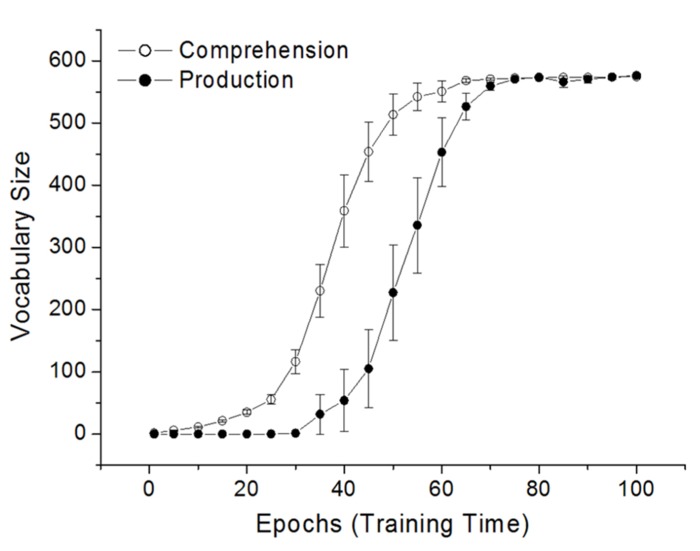
**Vocabulary spurt in the learning of the 591 CDI words by DevLex-II.** Results are averaged across 10 simulation trials. Error bars indicate standard error of the mean (figure adapted from Li et al., 2007, reproduced with permission from Wiley and Sons, Inc.).

**Figure 3** presents the average receptive and productive vocabulary sizes across the course of DevLex-II training, averaged across 10 simulation trials. The simulation results show a clear vocabulary spurt, preceded by a stage of slow learning and followed by a performance plateau. On average, the model’s productive vocabulary did not accelerate until about 35–40 epochs, one-third into the total training time, reflecting the model’s early protracted learning of the representations of word forms, meanings, and sequences, and their associative connections. Once the basic organization of the lexicon was acquired in terms of lexical and semantic categories and their associations, vocabulary learning accelerated, which occurred quickly after 40 epochs of training.

Although the figure shows only the results of the associative connections (form-to-meaning for comprehension, and meaning-to-sequence for production), the hit rates for these connections depended directly on the shape or precision of self-organization in the separate feature maps (see Figure 3 on Li et al., 2007). In other words, the period of rapid increase in vocabulary size may have been prepared by the network’s slow learning of the structured representation of phonemic sequence, word phonology, and word semantics, as well as its learning of the mappings between these characteristics of the lexicon. Once the basic structures were established on the corresponding maps, the associative connections between maps could be reliably strengthened to reach a critical threshold through Hebbian learning.

**Figure 3** also shows that the vocabulary spurt occurred for both production and comprehension, rather than being restricted to only one modality, consistent with empirical studies (Reznick and Goldfield, 1992). Previous empirical studies have largely focused on children’s word production, but a few researchers have also questioned whether a comprehension vocabulary spurt could exist. Our DevLex-II model was able to simulate a spurt pattern in both comprehension and production. Interesting, although both types of spurt were present in our simulations, the comprehension spurt occurred earlier than the production spurt, which is consistent with the argument that comprehension generally precedes production (Clark and Hecht, 1983) and in the case of lexical acquisition, a spurt in the receptive vocabulary could start much earlier (e.g., from 14 months of age; see Benedict, 1979).

Our simulation results as shown in **Figure 3** also indicated that there were significant individual differences between different simulation trials, even when all simulations had the same modeling parameters. Most interestingly, the largest variations tended to coincide with the rapid growth or spurt period. Examining the individual trials in detail, we found that different simulated networks could differ dramatically in the onset time of their vocabulary spurt. In the 10 simulation trials, the rapid increase of vocabulary size in production could begin from as early as epoch 30, or from as late as epoch 60, but in each case there was a clear spurt process. While some of these variations may be random effects (due primarily to the network’s random initial weight configurations and the random order of training words), others were systematic differences as a function of learning the complexity of the lexical input, especially the different stimulus properties such as word frequency and word length. Our simulation results clearly indicate the higher the word frequency, or the shorter the word length, the earlier the vocabulary spurt (see discussions in Li et al., 2007, Section 3.3).

#### Modeling early phonological production

DevLex-II is also able to simulate patterns in early phonological production. **Table 1** presents a list of typical examples from the same network discussed above on word productions at different training times. These errors parallel children’s early word pronunciations (Foster-Cohen, 1999), such as omission of consonants at the end of a word (e.g., output for “bib” at epoch 50), deletion of a consonant from consonant clusters (e.g., output for “smile” and “glue” at epoch 60), and substitution of consonants with similar phonemes (e.g., producing /b/ for /d/ in “bird”). These errors can be attributed to (a) incomplete meaning-to-phoneme links, and (b) incomplete sequence learning of phonemes. Our modeling results showed clearly how children’s early phonemic errors can arise from incomplete consolidation of word sequences, amplified by limitations in the learner’s phonemic memory.

**Table 1:** Sample production errors from DevLex-II in learning English vocabulary.

Words	pochs (training time)					
	30	40	50	60	80	100
Foot (/fυt/)	/υu:/	/fυ/	/fυ/	/fυ/	/fυt/	/fυt/
Dog (/dƆ:g/)	^–^	/d/	/d/	/dƆ:/	/dgƆ:/	/dƆ:g/
Sock (/sɑ:k/)	/ʃd/	/su:/	/su:/	/sɑ:/	/sɑ:/	/sɑ:/
Bib (/bɪb/)	/ɑ:/	/br/	/bɪ/	/bɪ/	/bɪb/	/bɪb/
Apple (/æpəl/)	^–^	/æp/	/æp/	/æp/	/æpə/	/æpəl/
Cat (/cæt/)	/a/	/cæ/	/cæ/	/cæ/	/cæt/	/cæt/
Brush (/brəʃ/)	/n/	/bən/	/bən/	/bəʃ/	/bəʃr/	/brəʃ/
Smile (/smaɪl/)	/pəɪi:/	/ɪma/	/ɪma/	/maɪl/	/maɪl/	/maɪl/
Glue (/glu:/)	/ɪ/	/g/	/g/	/gu:/	/gu:/	/gu:/
Sock (/sɑ:k/)	/a/	/sɑ:t/	/sɑ:t/	/sɑ:t/	/sɑ:t/	/sɑ:t/
Hide (/haɪd/)	/ɪb/	/ɪhb/	/haɪb/	/haɪb/	/haɪb/	/haɪb/
Bird (/bε:d/)	/υ(d;n)/^*^	/bb/	/bε:b/	/bε:b/	/bε:b/	/bε:b/
Bottle (/bɑ:təl/)	/tæ/	/btɑ:əæ/	/btɑ:əæ/	/btɑ:əæ/	/bɑ:təæ/	/bɑ:təæ/
Glasses (/glæsəz/)	/ts/	/æzi:n/	/gæzi:n/	/gæsəz/	/gæsəzl/	/glæsəz/

“–” indicates no unique output of the word since the word is confused with other words on the semantic map at the current time.

* Both the phonemes /d/ and /n/ on the phonemic map were the best matching units (BMUs) in response to the semantic representation of “bird.”

An examination of **Table 1** also indicates other interesting patterns. For example, in two different simulation trials, responding to the word *sock*, the network gave two different patterns of production error, the deletion of consonant /k/, and the substitution of it with /t/ (see **Table 1** for the two cases of *sock*). Given that the simulation trials had the same training parameters with the only difference in initial weights and training order of words, this difference reflects individual variations that are similar to those found both within and across different developmental stages in children (Menn and Stoel-Gammon, 1993).

Finally, most of the examples in **Table 1** also reflect a general developmental shift in phonological pattern formation. At the earliest stages of learning, the network’s productions were highly simplified and often very different from the target pronunciations. During the middle and late stages of learning, with the emergence of self-organized phonemic structure and the developing associative links, correct productions increased gradually. At these stages, the production errors, though still present, were much closer to the target pronunciations but some also had typical error patterns as discussed above. The coexistence of correct and incorrect word pronunciations corresponds to empirical patterns in children’s phonological development from babbling to word production (Menn and Stoel-Gammon, 1993; Foster-Cohen, 1999). The transition from incorrect sequences, omissions, and substitutions to correct pronunciations indicates that our model was able to capture developmental changes in early phonological acquisition.

#### Modeling the acquisition of grammatical and lexical aspect

Linguists generally distinguish between two kinds of aspect, grammatical aspect and lexical aspect. Grammatical aspect is related to aspectual distinctions which are often marked explicitly by linguistic devices (e.g., English auxiliary *be* plus inflectional suffix *ing* to mark ongoing activities). Lexical aspect, on the other hand, refers to the characteristics inherent in the temporal meanings of a verb, for example, whether the verb encodes an inherent end point of a situation (e.g., telic verb like *arrive* vs. atelic verb like *run*), or whether the verb is inherently stative or punctual (stative verb like *believe* vs. punctual verb like *break*). Research has shown that there is a strong interaction between these two types of aspect in the process of children’s early lexical and grammatical acquisition (see Li and Shirai, 2000 for a review); for example, initially children’s use of grammatical inflections is restricted to specific verbs (e.g., using -*ed* only with punctual verbs), and only later on it approaches the adult linguistic pattern.

We wanted to see whether a multi-layer SOM-based model is able to capture the developmental patterns of child language in the acquisition of lexical and grammatical aspect, and whether a connectionist network void of pre-specified categories can acquire verb aspectual categories that have been claimed to be innate (cf. the “language bioprogram hypothesis” of Bickerton, 1984). To simulate the acquisition of aspect, DevLex-II was trained on 184 English verbs across four growth age stages (or input ages: 13–18, 19–24, 25–30, and 31–36 months old). Each of the 184 verb types was chosen if it occurred in the parental speech of CHILDES database (MacWhinney, 2000) for 50 or more times within a certain age group mentioned above (see Zhao and Li, 2009 for details). We examined the network’s acquisition of imperfective/progressive aspect marker *ing*, habitual aspect markers *-s* and perfective aspect marker *-ed* in connection with the acquisition of three semantic categories of lexical aspects (*activity*, *telic*, and *stative* verbs). Here, we defined the correct production of the aspect form for any given verb in the same way as done in Li et al. (2007): for example, if the word *kicking* is shown to the semantic map, production is counted correct only when the consecutively activated nodes on the output phonemic map are the BMUs for /k/ /I/ /k/ /I/ /η/ in the correct sequence.

**Table 2** presents a comparison of our simulation results with empirical patterns from parental speech. First, looking at the simulation data, we found that the network’s production of inflectional markers across the four input ages are highly consistent with the empirical patterns: the use of imperfective aspect (*-ing*) is closely associated with activity verbs that indicate ongoing process, while the use of perfective aspect (*-ed*) is closely associated with telic verbs that indicate actions with endpoints or end results. In particular, in early child English, *-ing* is highly restricted to activity verbs, *-ed* restricted to telic verbs, and *-s* restricted to stative verbs, as demonstrated by Bloom et al. (1980). Our network, having received input patterns based on parental speech from the CHILDES database, behaved in the same way as children do. For example, at input age 1;6, the network produced *-ing* predominantly with activity verbs (73%), *-ed* overwhelmingly with telic verbs (75%), and *-s* with stative verbs (57%). Such associations were observed at all four stages (especially for *-ing* and *-ed*), but they became attenuated over time.

**Table 2:** Percentage of use of tense-aspect suffixes with different verb types across input age groups in DevLex-II's production and in input data based on parental speech (adapted from Li and Zhao, 2009, reproduced with permission from Mouton de Gruyter).

Verbs	Tense-aspect suffixes											
	Age 1;6			Age 2;0			Age 2;6			Age 3;0		
	*-ing*	*-ed*	*-s*	*-ing*	*-ed*	*-s*	*-ing*	*-ed*	*-s*	*-ing*	*-ed*	*-s*
**Network production**
Activity	73	0	29	69	27	33	61	24	35	62	30	37
Telic	27	75	14	21	53	28	32	62	27	31	62	26
Stative	0	25	57	10	20	39	7	14	38	7	8	37
**Parental input data**
Activity	63	23	29	62	26	26	63	22	33	60	29	35
Telic	31	62	29	31	58	26	29	66	25	32	59	24
Stative	6	15	43	7	16	48	8	12	42	8	12	41

Second, we analyzed the input dataset to our network (based on child-directed parental speech), and found that there was also a clear consistency between the input and the network’s production. In the input data there are clear associations between *-ing* and activity verbs, *-ed* and telic verbs, and that these associations are strong throughout the four input ages, as shown also by Shirai (1991) in an empirical analysis. The degree to which the network’s production matches up with the input patterns indicates that DevLex-II was able to capture the statistical co-occurrences relationship between lexical aspect (verb types) and grammatical aspect (verb morphology) in the input. While this is hardly surprising for a connectionist model, our results also indicate that DevLex-II’s productions were not simply verbatim mimics of what’s in the input by recording individual words and suffixes and their co-occurrence. This is important in that our network has derived (but not simply reproduced) the type–suffixes association patterns from the linguistic input. The simulation results showed that the associations between verb types and suffixes were stronger in the network’s productions than they were in the input data received by the network, particularly for the early training stages (i.e., more restrictive associations between verb semantics and inflectional suffixes, for example, between telic verbs and *-ed*). DevLex-II at early stages behaved more restrictively than what is in the language input with respect to the correlations between lexical aspect and grammatical aspect, which matches up well with empirical observations from child language (see Li and Shirai, 2000 for review).

### DevLex-II MODELS OF BILINGUAL LANGUAGE ACQUISITION

While the above discussion highlighted two domains (vocabulary and grammatical morphology) in which DevLex-II was applied to first language (L1) acquisition (see Zhao and Li, 2013 for a full list of DevLex-II applications), the utility of the model as a general model of language acquisition has also been tested further in the study of second language (L2) acquisition. Below we discuss how DevLex-II has been applied to examine a range of key issues in bilingualism.

#### Modeling age-of-acquisition effects

Much of the current debate about the nature of L2 learning and how it differs from L1 learning stems from the “critical period” hypothesis. Indeed, interests in the critical period hypothesis have led *Science* magazine in its 125th anniversary issue to designate the understanding of critical periods of language acquisition as one of the top 125 big science questions in all scientific domains of inquiry for the next quarter century (*Science*, vol. 309, July 1, 2005). Recent studies, however, have argued against the original account of Lenneberg (1967) that there is a biologically based critical period for language acquisition due to brain lateralization; instead, the evidence points to cognitive and linguistic mechanisms underlying the AoA effects seen with both L1 and L2 acquisition (see MacWhinney, 2012; Li, in press). For example, Johnson and Newport (1989) suggested that language learning in childhood confers certain cognitive advantages precisely because of the child’s limited memory and cognitive resources (the “less is more” hypothesis; see also Elman, 1990). Hernandez and Li (2007) suggested that different sensorimotor processing and control characteristics could underlie child vs. adult learning and processing differences (the “sensorimotor integration hypothesis”; see also Bates, 1999). Finally, MacWhinney (2012) suggested that certain risk factors (e.g., entrenchment of L1, negative transfer, and social isolation) with late learners but not early learners could be responsible for the age-related learning effects in language acquisition (the “unified competition model” hypothesis).

In an effort to provide computational insights into the AoA effects, Zhao and Li (2010) applied the DevLex-II model to 1000 words, 500 in Chinese as L1 and 500 in English as L2, selected from the CDI database (Dale and Fenson, 1996). These words were presented to the model systematically in three different learning scenarios: simultaneous learning of L1 and L2; early L2 learning; and late L2 learning. For simultaneous learning, the two lexicons were presented to the network and trained in parallel (see Li and Farkavs, 2002 for a previous example in this training regime). For early L2 learning, the onset time of L2 input to the model was slightly delayed relative to that of L1 input, that is, training on L2 vocabulary occurred at a point after 1/5 of the entire L1 vocabulary had been presented to the network. For late L2 learning, the onset time of L2 input was significantly delayed relative to that of L1, that is, training on L2 vocabulary occurred at a point after 4/5 of the entire L1 vocabulary had been presented to the network. Specifically, the simultaneous learning situation is analogous to a situation in which children are raised in a bilingual family and receive linguistic inputs from the two languages simultaneously (e.g., Li and Farkavs, 2002 used input based on the two parents’ different language input). The early learning situation could be compared to the situation in which bilinguals acquired their L2 early in life (e.g., in early childhood) while the late learning situation to that of a bilingual’s learning of L2 later in life (e.g., after puberty).

**FIGURE 4:**
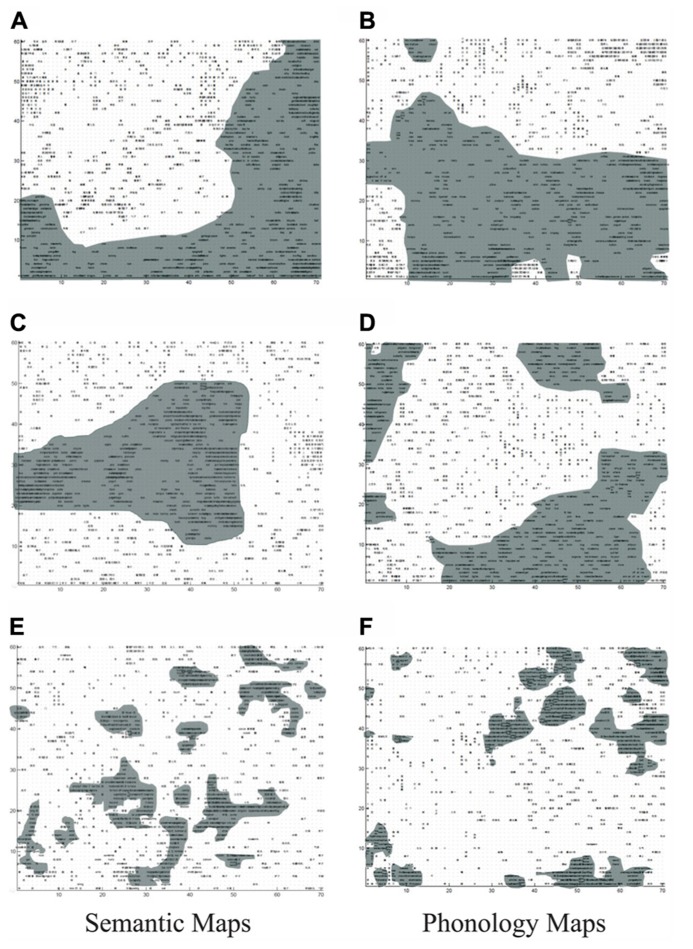
**Bilingual lexical representation of semantics and phonology respectively as a function of age of acquisition (AoA).** Dark areas correspond to L2 (English) words. **(A,B)** Simultaneously learning; **(C,D)** early L2 learning; **(E,F)** late L2 learning.

One key pattern from our simulations is illustrated in **Figure 4**, which shows how lexical representations from the two languages are distributed differently in the three different learning conditions. Here, black regions indicate those nodes that represent the L2 (English) words whereas white regions the L1 (Chinese) words learned by the model. Specifically, if a unit’s weight vector is the closest to the input vector of an English word, the unit is marked in black. If a unit’s weight vector is most similar to the input pattern of a Chinese word, the unit is marked in white.

It is clear from **Figure 4** that the relative onset time of L2 vs. L1 plays an important role in modulating the overall representational structure of the L2^6^. For the simultaneous acquisition situation (**Figures 4A,B**), DevLex-II showed clear distinct lexical representations of the two languages at both the phonological and semantic levels and within each language. The results suggest that simultaneous learning of two languages allows the system to easily separate the lexicons during learning, consistent with the simulation patterns from Li and Farkavs (2002). In the case of sequential acquisition, if L2 was introduced into learning early on, the lexical organization patterns were similar (though not identical) to those found in simultaneous acquisition, as shown in **Figures 4C,D**. The differences were in terms of the slightly smaller spaces occupied by the L2 words (English) as compared to the lexical space occupied by L1^7^. The L2 lexicon was still able to establish its separate territory of lexical representation. However, if L2 was introduced to learning at a late stage, the lexical organization patterns were significantly different from those found in simultaneous acquisition, as shown in **Figures4E,F**. No large independent areas for the L2 representation appeared this time. Indeed, the L2 representations appeared to be parasitic or auxiliary to those of L1 words: compared with L1 words, the L2 words occupied only small and fragmented regions, and were dispersed throughout the map. There were small L2 chunks that were isolated from each other, and interspersed within L1 regions. Interestingly, the parasitic nature of the L2 representation is also reflected in the locations of the L2 words in the map, which was determined by the similarity of the L2 words to the established L1 words in meaning (for semantic map) or in sound (for phonological map).

The biologically based account of a critical period as originally put forth by Lenneberg (1967) is intuitively appealing, but the modeling results presented here indicate that we can simulate critical period-like effects without invoking any significant changes in the architecture or mechanisms in the network. A significant contribution of connectionist models to the understanding of development, according to Elman et al. (1996), is that these models do not involve pre-determined or pre-specified categories or underlying differences, and yet the simulated data show that categories or differences in these models emerge as a result of learning itself across a developing learning history. The “age” effects that were simulated in our model may reflect the changing dynamics inherent in learning and the interactions between the two languages across different types of learning situation. The idea that the learning process itself can lead to differences in the dynamics and outcomes of development is not new (see Elman et al., 1996; Thomas and Johnson, 2008).

What is new from our studies is that the age-related effects, traditionally attributed to inherent properties of the learner, emerged in our models as a result of learning the same L2 targets at different time points of learning. The effects of dynamic interactions in the two competing languages clearly speak for the perspective of competition, entrenchment, and plasticity in accounting for critical period effects (see General Discussion for more discussion).

#### Modeling cross-language priming

One important goal of simulation is to provide a mechanistic account of the observed behavioral phenomena found in empirical studies (see, for example, Richardson and Thomas, 2008 for discussion). Capitalizing on the above findings of the impact of simulated age effects on bilingual lexical organization, Zhao and Li (2013) extended DevLex-II to simulate cross-language semantic priming in connection with the AoA effect. Cross-language priming has been a vital empirical method in the literature for testing semantic representations in bilinguals, and many studies have shown that in such a paradigm bilinguals respond faster to translation equivalents or semantically related words across languages than to unrelated pairs of words from the two languages (named as *translation priming* and *semantic priming*, respectively). Zhao and Li (2013) implemented a spreading activation mechanism in DevLex-II so that cross-language priming could be modeled. This mechanism involves two parts: (1) nodes on a map were fully connected with each other via lateral connections, and their weights were trained via Hebbian learning, triggered by the joint presentations of translation equivalents. This type of associative connections was added to DevLex-II specifically for modeling priming effects; (2) spreading activation from a prime word to a target word could occur via two paths, one through the lateral connections and one within the semantic map^8^.

**FIGURE 5:**
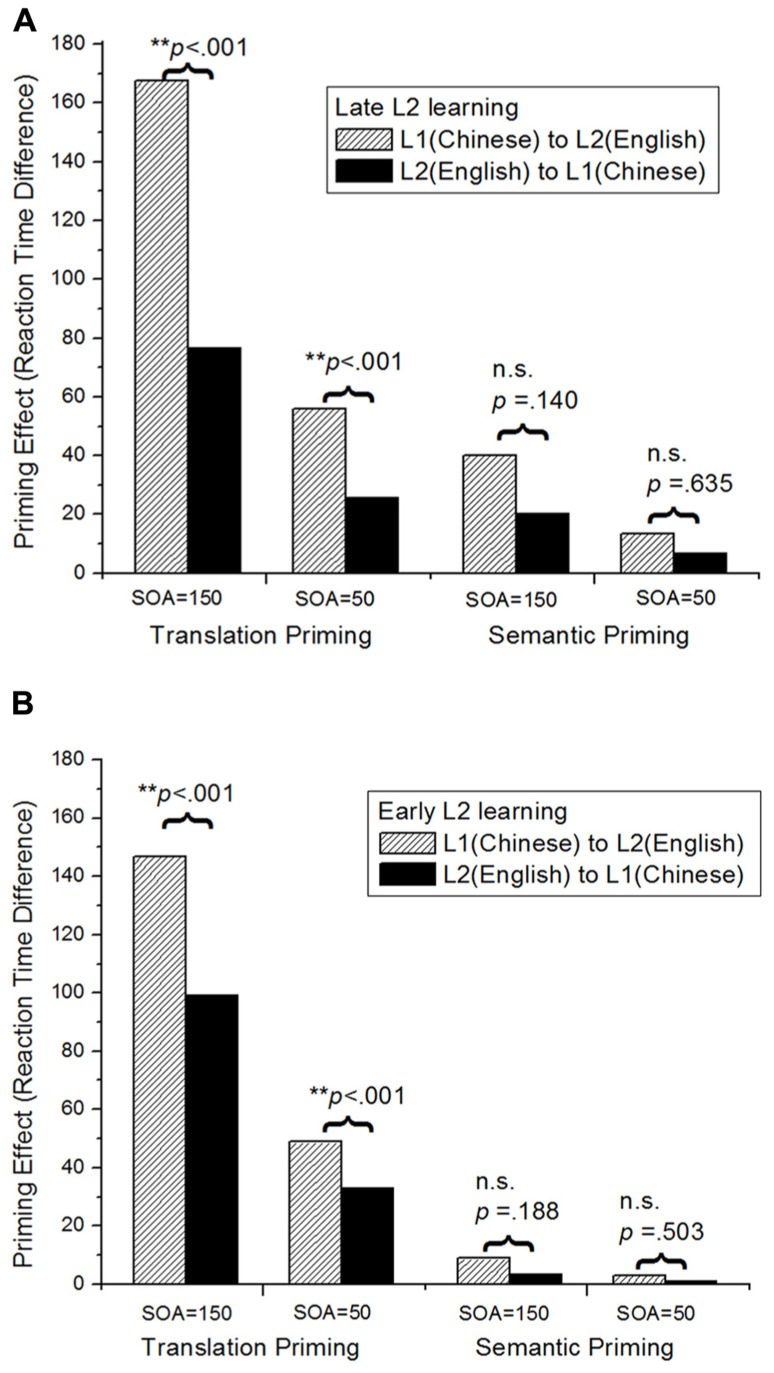
**Cross-language priming and priming asymmetry effects shown in DevLex-II: (A)** late L2 learning; **(B)** early L2 learning. Priming effects are calculated by subtracting the RTs of related word pairs from the RTs of unrelated word pairs. SOA represents the stimulus onset asynchrony. The *p* values indicate the significance level of the priming asymmetry under the different conditions (paired-samples *t*-test of the 20 simulations under each condition: **significant priming asymmetry; n.s., not significant) (adapted from Zhao and Li, 2013, reproduced with permission from Cambridge University Press).

**Figure 5** presents the basic results of our simulations. The model clearly displayed both translation priming and semantic priming effects, although translation priming was always stronger than semantic priming, consistent with patterns from empirical studies (Basnight-Brown and Altarriba, 2007). Another important simulated pattern was the “priming asymmetry”: in the empirical literature (see Dimitropoulou et al., 2011 for a review), it has been observed that priming effects are stronger if participants are presented with L1 words as primes and L2 words as targets (i.e., the L1-to-L2 direction of priming), as compared with the situation in which participants are presented with L2 words as primes and L1 words as targets (i.e., the L2-to-L1 direction of priming). As seen in **Figure 5**, the priming effects from L1 (Chinese) to L2 (English) were always larger than those from L2 to L1. More interestingly, such “priming asymmetry” decreased as a function of the effect of AoA; for example, it was larger in the late L2 learning situation than in the early L2 learning situation.

DevLex-II provided a mechanistic account for this asymmetry, following the ideas discussed above regarding AoA effects, by reference to the richness of semantic representation of the L2 in our model (i.e., the number of activated semantic features that will lead to different degrees of priming from L2 primes to L1 targets). This account is particularly significant in light of the DevLex-II’s emphasis on cross-language lexical competition. Specifically, the richness of semantic representation and the potential lexical competition are inversely related: the richer or more elaborated the representation of a word, the less competition (and hence less confusion) the learner may experience between the word and other lexical items in the memory. If the priming is from the L2 to L1 direction, due to the dense representation of L2 (thus strong competition), a brief exposure to L2 may not trigger initial activations strong enough to spread to the target L1 items not directly adjacent in the representation. In contrast, activations of L1 items could be much stronger given that they are more sparsely represented. If the priming is from the L1 to L2 direction, it will be always larger than the reverse, due to distinct (and richer) semantic representations of the L1 words (thus having less competition). Consequently, the amount of priming from L2 to L1 (and L1 to L2) may be enhanced or decreased, depending on a bilingual’s L2 level as a function of AoA or proficiency, thereby giving rise to the different amount of “priming asymmetry.” If the L2 is acquired at an early stage, its semantic representations are more enriched, and more distinct from L1 representations (rather than depending or being parasitic on L1 representations, as discussed earlier). In this case, the L2 to L1 priming effects are more comparable to the L1 to L2 priming effects given the similar representations of the two lexicons, thus cause a less salient priming asymmetry. Such an account has found empirical support in the semantic priming literature, in both the L1 and the L2 context (see discussions in Wang and Forster, 2010; Dimitropoulou et al., 2011).

## GENERAL DISCUSSION

In this article, we first reviewed previous connectionist models based on SOMs, and discussed the significant implications that SOM-based models have for understanding language representation and acquisition. We then described a specific model using SOM in the study of language acquisition, the DevLex-II model. We presented an overview of how this model can be successfully used to address a number of important issues in monolingual and bilingual language acquisition, and illustrated its properties and applications in several psycholinguistic domains, including the modeling of vocabulary spurt, aspect acquisition, AoA effects, and cross-language semantic priming. We demonstrated that DevLex-II is a scalable model that can account for a variety of linguistic patterns in child and adult language learning.

We can highlight here a few key features of DevLex-II for the study of language acquisition. First, in contrast to previous computational models, DevLex-II is based on unsupervised learning (specifically SOM) and Hebbian learning, two powerful and biologically plausible principles of computation. These principles have allowed us to simulate the dynamics underlying both monolingual and bilingual lexical representations and interactions. Second, DevLex-II relies on the use of large-scale realistic linguistic data as the input. By simulating actual lexical forms and meanings, we are able to achieve developmental and lexical realism in our models. Third, DevLex-II incorporates computational learning properties (e.g., self-adjustable neighborhood functions, spreading activation, lateral connection) against the context of realistic language learning so that it can be used to simulate both language acquisition and language processing, in both L1 and L2 contexts.

To scholars of monolingual language acquisition, connectionist learning models are no new beasts. The original Rumelhart and McClelland (1986) past tense model and the subsequent debates, the Elman et al. (1996) book on rethinking innateness, and the more recent special issue edited by MacWhinney (2010) have all popularized the utility of connectionist models. Most researchers in L1 studies can appreciate the distinct advantages of connectionist learning models in allowing us to manipulate variables of interest flexibly and to study their interactions in a more systematic way (e.g., input quantity and quality, word frequency, word length in affecting error patterns). However, to scholars of bilingual language acquisition, the utility of connectionist models has yet to be fully appreciated.

The most popular computational model in bilingualism, the Bilingual Interactive Activation (BIA) model (Dijkstra and van Heuven, 1998), was based on the interactive activation (IA) model of McClelland and Rumelhart (1981). IA-based models typically lack a learning mechanism, and as such, they tend to focus on capturing representation and processing states of mature bilingual speakers and listeners (which is important in its own right). Computationally implemented learning models of bilingualism, however, remain scarce. It is important for researchers to develop connectionist learning models to capture the acquisition and interaction of multiple languages. This is because through modeling we can systematically identify the interactive effects of the two languages in terms of L2 onset time, L2 input frequency, amount of L1 vs. L2 input, order of L1 vs. L2 learning, and how these variables may separately or jointly impact both the learning trajectory and the learning outcome.

In a recent special issue edited by Li (2013) on computational modeling of bilingualism, a number of studies have attempted to fill the gap by taking advantage of the features of connectionist models to study bilingual acquisition and processing (e.g., Cuppini et al., 2013; Monner et al., 2013; Zhao and Li, 2013). These studies not only attempted to address specific problems and disentangle the effects of entrenchment, proficiency, memory resources, and lexical semantic distances, but also provided mechanistic accounts of important theoretical issues. For example, Monner et al. (2013) tested specifically the “less is more” hypothesis (Johnson and Newport, 1989) in a connectionist model, in which the increase of working memory was simulated by the use of new cell assemblies in the model, whereas L1 entrenchment was simulated by the training of the network with variable-length exposure of L1 before the onset of L2. In this way, the modeling results allowed the researchers to dissociate effects due to the increase of memory and the increase of age, which are confounded in natural learning settings.

Monner et al.’s (2013) model illustrates the important role that connectionist modeling can play in second language learning, and at the same time speaks to the possibility that age-related learning differences as prescribed by the critical period hypothesis may be accounted for by the interactive effects of entrenchment of L1 and computational resources, which is highly consistent with the simulation results from DevLex-II as discussed above (see also Hernandez et al., 2005). The degree of entrenchment is a result of how well established the network has the L1 representation structure: the more consolidated the representation (as in late L2 learning), the more resistant to change the topographic structure becomes in the model. New items from the L2 have to use existing structures built from the L1, and any further learning is only able to result in what we call “parasitic” representations. By contrast, when learning occurs early, fewer L1 words may have become fully consolidated in the representation and the network may be less committed to L1 representations, and therefore the system is still open to adaptation in the face of new input from L2 so as to be able to continually reorganize and restructure the L2 representations. It is important to note that timing itself is not the cause, but time of learning is accompanied by different dynamics of interactions between the two languages for learning. Simulations from Monner et al.’s (2013) model and from DevLex-II suggest that the nature of bilingual representation is the result of a highly dynamic and competitive process in which early learning constrains later development, therefore shaping the time course and structure of later language systems. To what extent early learning impacts later learning, and to what extent extensive later learning can soften or even reverse early-learned structure, will remain the key research questions in the years to come.

What would be the future for SOM-based connectionist language models, in particular the DevLex-II model? One issue to bear in mind as we move forward is that we must bridge computational modeling results with a variety of other behavioral, neuropsychological, and neuroimaging findings, especially given the neurally plausible architectures of multiple SOM models (e.g., DevLex-II or DISLEX). As discussed earlier, Kiran et al. (2013) provided an excellent example in this regard, in which the investigators constructed a model to simulate neuropsychological patterns of each of 17 bilingual patients following traumatic brain injury and subsequent treatment. A second dimension to explore SOM-based models for language acquisition is to further identify the relationship between map organizations developed at different stages of learning and the impact that these different organizations may have on the behavior of learning (e.g., in terms of speed and outcome of learning success). DevLex-II has made some efforts in this regard, for example, in simulating vocabulary spurt and cross-language semantic priming, by linking the representational structure and semantic richness of the representation to the performance (e.g., word learned or priming effects) in the model, but more needs to be done. A third dimension to extend SOM-based models of language may be to study how syntactic structures can be acquired in both L1 and L2. Given the status of syntax in linguistic theories, connectionist models have yet to demonstrate their utility in learning syntactic structures. Elman (1990) showed that the simple recurrent network (SRN) can learn the hierarchical recursive structure of sentences. One could consider to introduce mechanisms into SOMs to capture temporal order information in language representation by using recursive SOMs (see Tiňo et al., 2006 for an example).

As we think ahead we also must develop SOM models of language that can make distinct predictions in light of the simulations and empirical data. In some cases, the empirical data may have not yet been obtained, or cannot be obtained (e.g., as in the case of brain injury, one cannot go back to pre-lesion conditions), and this is the occasion where modeling results will be extremely helpful. Not only should computational modeling verify existing patterns of behavior on another platform, it should also inform theories of L1 and L2 acquisition by making distinct predictions under different hypotheses or conditions. In so doing, computational modeling will provide a new forum for generating novel ideas, inspiring new experiments, and helping formulate new theories (see McClelland, 2009 for a discussion of the role of modeling in cognitive science). Finally, computationally minded researchers in language science should follow a recent call by Addyman and French (2012) to make an effort to provide user-friendly interfaces and tools to non-modelers, so that many more students of language acquisition can test computational models without fearing the technical hurdles posed by programming languages, source codes, and simulating environments.

## Conflict of Interest Statement

The authors declare that the research was conducted in the absence of any commercial or financial relationships that could be construed as a potential conflict of interest.

## Footnotes

3 There are other connectionist models that have neither PDP nor SOM architectures that have been applied to language studies (see Davis, 1999; Bowers, 2002; see Li and Zhao, 2012 for a bibliography).

4 These are based on our modeling experience and the modeler needs to consider their utility in light of the task and complexity of input–output relationships in each simulation.

5 Based on this method, Zhao etal. (2011) developed a software (Contextual Self-Organizing Map Package) to derive corpus-based semantic representations in multiple languages using word co-occurrence statistics.

6 At this point we consider such segregations in the representation between L1 and L2 at the lemma level rather than a deeper semantic level, given the complexity associated with semantic and conceptual relations across languages (see Pavlenko, 2009 for a discussion).

7 Similar results were obtained when English was trained as the L1 and Chinese the L2.

8 Zhao and Li (2013) also developed a mechanism to capture the time elapses in lexical decision tasks so that differences in reaction time (RT) could be modeled for cross-language priming (see technical details in that paper).

## References

[B1] Addyman C. French R. M. (2012). Computational modeling in cognitive science: a manifesto for change. *Top. Cogn. Sci.* 4 332–341 10.1111/j.1756-8765.2012.01206.x 22711233

[B2] Basnight-Brown D. Altarriba J. (2007). Differences in semantic and translation priming across languages: the role of language direction and language dominance. *Mem. Cogn.* 35 953–965 10.3758/BF03193468 17910179

[B3] Bates E. (1999). “Plasticity, localization and language development,” in *The Changing Nervous System: Neurobehavioral Consequences of Early Brain Disorders* eds Broman S. Fletcher J. M. (New York: Oxford University Press) 214–253

[B4] Bates E. Carnevale G. (1993). New directions in research on language development. *Dev. Rev.* 13 436–470 10.1006/drev.1993.1020

[B5] Benedict H. (1979). Early lexical development: comprehension and production. *J. Child Lang.* 6 183–200 10.1017/S0305000900002245 468932

[B6] Bickerton D. (1984). The language bioprogram hypothesis. *Behav. Brain Sci.* 7 173–188 10.1017/S0140525X00044149

[B7] Bloom K. Lifter K. Hafitz J. (1980). Semantics of verbs and the development of verb inflection in child language. *Language* 56 386–412

[B8] Bowers J. S. (2002). Challenging the widespread assumption that connectionism and distributed representations go hand-in-hand. *Cogn. Psychol.* 45 413–445 10.1016/S0010-0285(02)00506-6 12480480

[B9] Clark E. V. (2009). *First Language Acquisition*, 2nd Edn. Cambridge: Cambridge University Press. 10.1017/CBO9780511806698

[B10] Clark E. V. Hecht B. F. (1983). Comprehension, production, and language acquisition. *Annu. Rev. Psychol.* 34 325–349 10.1146/annurev.ps.34.020183.001545

[B11] Cuppini C. Magosso E. Ursino M. (2013). Learning the lexical aspects of a second language at different proficiencies: a neural computational study. *Biling. Lang. Cogn.* 16 266 10.1017/S1366728911000617

[B12] Dale P. S. Fenson L. (1996). Lexical development norms for young children. *Behav. Res. Methods Instrum. Comput.* 28 125–127 10.3758/BF03203646

[B13] Davis C. (1999). *The Self-Organising Lexical Acquisition and Recognition (SOLAR) Model of Visual Word Recognition*. Unpublished doctoral dissertation, University of New South Wales Kensington

[B14] Dijkstra T van Heuven W. J. B. (1998). “The BIA model and bilingual word recognition,” in *Localist Connectionist Approaches to Human Cognition* eds Grainger J. Jacobs A. M. (Mahwah, NJ: Erlbaum) 189–226

[B15] Dimitropoulou M. Duñabeitia J. A. Carreiras M. (2011). Two words, one meaning: evidence of automatic co-activation of translation equivalents. *Front. Psychol.* 2:188 10.3389/fpsyg.2011.00188 PMC3155883 21886634

[B16] Elman J. (1990). Finding structure in time. *Cogn. Sci.* 14 179–211 10.1207/s15516709cog1402_1

[B17] Elman J. Bates E. A. Johnson M. H. Karmiloff-Smith A. Parisi D. Plunkett K. (1996). *Rethinking Innateness: A Connectionist Perspective on Development*. Cambridge, MA: MIT Press

[B18] Foster-Cohen S. H. (1999). *An Introduction to Child Language Development*. London: Longman

[B19] French R. M. (1999). Catastrophic forgetting in connectionist networks. *Trends Cogn. Sci.* 3 128–135 10.1016/S1364-6613(99)01294-2 10322466

[B20] Goldfield B. A. Reznick J. S. (1990). Early lexical acquisition: rate, content, and the vocabulary spurt. *J. Child Lang.* 17 171–183 10.1017/S0305000900013167 2312640

[B21] Grossberg S. (1976a). Adaptive pattern classification and universal recoding: I. Parallel development and coding of neural feature detectors. *Biol. Cybern.* 23 121–134 10.1007/BF00344744 974165

[B22] Grossberg S. (1976b). Adaptive pattern classification and universal recoding: II. Feedback, expectation, olfaction, illusions. *Biol. Cybern.* 23 187–202 10.1007/BF00340335 963125

[B23] Guenther F. H. Gjaja M. N. (1996). The perceptual magnet effect as an emergent property of neural map formation. *J. Acoust. Soc. Am.* 100 1111–1121 10.1121/1.416296 8759964

[B24] Haykin S. (1999). *Neural Networks: A Comprehensive Foundation*, 2nd Edn. Upper Saddle River, NJ: Prentice Hall

[B25] Hebb D. (1949). *The Organization of Behavior: A Neuropsychological Theory*. New York: Wiley

[B26] Hernandez A. Li P. (2007). Age of acquisition: its neural and computational mechanisms. *Psychol. Bull.* 133 638 10.1037/0033-2909.133.4.638 17592959

[B27] Hernandez A. Li P. MacWhinney B. (2005). The emergence of competing modules in bilingualism. *Trends Cogn. Sci.* 9 220–225 10.1016/j.tics.2005.03.003 15866148 PMC4107303

[B28] Hinton G. E. Sejnowski T. J. (1999). *Unsupervised Learning: Foundations of Neural Computation*. Cambridge, MA: The MIT press

[B29] James D. Miikkulainen R. (1995). “SARDNET: a self-organizing feature map for sequences,” in *Advances in Neural Information Processing Systems* Vol.7 eds Tesauro G. Touretzky D. S. Leen T. K. (Cambridge, MA: MIT Press) 577–584

[B30] Johnson J. S. Newport E. L. (1989). Critical period effects in second language learning: the influence of maturational state on the acquisition of English as a second language. *Cogn. Psychol.* 21 60–99 10.1016/0010-0285(89)90003-0 2920538

[B31] Kiran S. Graesman U. Sandberg C. Miikkulainen R. (2013). A computational account of bilingual aphasia rehabilitation. *Biling. Lang. Cogn.* 16 325 10.1017/S1366728912000533 PMC3940390 24600315

[B32] Kohonen T. (2001). *Self-Organizing Maps* 3rd Edn Berlin: Springer. 10.1007/978-3-642-56927-2

[B33] Kuhl P. K. (1991). Human adults and human infants show a “perceptual magnet effect” for the prototypes of speech categories, monkeys do not. *Percept. Psychophys.* 50 93–107 10.3758/BF03212211 1945741

[B34] Lenneberg E. H. (1967). *Biological Foundations of Language*. New York, NY: Wiley

[B35] Li P. (2003). “Language acquisition in a self-organising neural network model,” in *Connectionist Models of Development: Developmental Processes in Real and Artificial Neural Networks* ed. Quinlan P. (Hove: Psychology Press) 115–149

[B36] Li P. (in press). “Bilingualism as a dynamic process,” in *Handbook of Language Emergence* eds MacWhinney B. O'Grady W. (Boston: John Wiley & Sons, Inc.)

[B37] Li P. (2013). Computational modeling of bilingualism. *Biling. Lang. Cogn.* 16 241–366 10.1017/S1366728913000059

[B38] Li P. Burgess C. Lund K. (2000). “The acquisition of word meaning through global lexical co-occurrences,” in *Proceedings of the Thirtieth Annual Child Language Research Forum* ed. Clark E. V. (Stanford, CA: Center for the Study of Language and Information) 167–178

[B39] Li P. Farkaš I. (2002). A self-organizing connectionist model of bilingual processing. *Adv. Psychol.* 134 59–85 10.1016/S0166-4115(02)80006-1

[B40] Li P. Farkaš I. MacWhinney B. (2004). Early lexical development in a self-organizing neural network. *Neural Netw.* 17 1345–1362 10.1016/j.neunet.2004.07.004 15555870

[B41] Li P. MacWhinney B. (2002). PatPho: a phonological pattern generator for neural networks. *Behav. Res. Methods Instrum. Comput.* 34 408–415 10.3758/BF03195469 12395557

[B42] Li P. Shirai Y. (2000). *The Acquisition of Lexical and Grammatical Aspect*. Berlin: Mouton de Gruyter

[B43] Li P. Zhao X. (2009). “Computational modeling of the expression of time,” in *The Expression of Time* eds Klein W. Li P. (Berlin: Mouton de Gruyter) 241–271

[B44] Li P. Zhao X. (2012). “Connectionism,” in *Oxford Bibliographies Online: Linguistics* ed. Aronoff M. (New York, NY: Oxford University Press). Available at:

[B45] Li P. Zhao X. MacWhinney B. (2007). Dynamic self-organization and early lexical development in children. *Cogn. Sci.* 31 581–612 10.1080/15326900701399905 21635309 PMC9808815

[B46] MacWhinney B. (2000). *The CHILDES Project: Tools for Analyzing Talk*. Hillsdale, NJ: Lawrence Erlbaum

[B47] MacWhinney B. (2010). Computational models of child language learning: an introduction. *J. Child Lang.* 37 477–485 10.1017/S0305000910000139 20420743 PMC4070657

[B48] MacWhinney B. (2012). “The logic of the unified model,” in *Routledge Handbook of Second Language Acquisition* eds Gass S. Mackey A. (New York, NY: Routledge) 211–227

[B49] Markman E. M. (1994). Constraints on word meaning in early language acquisition. *Lingua* 92 199–227 10.1016/0024-3841(94)90342-5

[B50] Mayor J. Plunkett K. (2010). A neurocomputational account of taxonomic responding and fast mapping in early word learning. *Psychol. Rev.* 117 1–31 10.1037/a0018130 20063962

[B51] McClelland J. (2009). The place of modeling in cognitive science. *Top. Cogn. Sci.* 1 11–28 10.1111/j.1756-8765.2008.01003.x 25164798

[B52] McClelland J. Rumelhart D. (1981). An interactive activation model of context effects in letter perception: part 1. An account of basic findings. *Psychol. Rev.* 88 375–407 10.1037/0033-295X.88.5.375 7058229

[B53] McClelland J. Rumelhart D the PDP Research Group (1986). *Parallel Distributed Processing: Explorations in the Microstructure of Cognition*, Vol. 2. Cambridge, MA: MIT Press 10.1111/cogs.12148 25087578

[B54] Menn L. Stoel-Gammon C. (1993). “Phonological development: learning sounds and sound patterns,” in *The Development of Language* 3rd Edn ed. Gleason J. B. (New York, NY: Macmillan) 65–113

[B55] Mervis C. B. Bertrand J. (1994). Acquisition of the novel name-nameless category (N3C) principle. *Child Dev.* 65 1646–1663 10.2307/1131285 7859547

[B56] Miikkulainen R. (1993). *Subsymbolic Natural Language Processing: An Integrated Model of Scripts, Lexicon, and Memory*. Cambridge, MA: MIT Press

[B57] Miikkulainen R. (1997). Dyslexic and category-specific aphasic impairments in a self organizing feature map model of the lexicon. *Brain Lang.* 59 334–366 10.1006/brln.1997.1820 9299068

[B58] Miikkulainen R. Bednar J. A. Choe Y. Sirosh J. (2005). *Computational Maps in the Visual Cortex*. New York: Springer

[B59] Miikkulainen R. Kiran S. (2009). “Modeling the bilingual lexicon of an individual subject,” in *Lecture Notes in Computer Science 5629: Proceedings of the Workshop on Self-Organizing Maps* (WSOM'09, St. Augustine, FL) (Berlin: Springer), PMC2767190

[B60] Miller G. A. (1990). WordNet: an on-line lexical database. *Int. J. Lexicogr.* 3 235–312 10.1093/ijl/3.4.235

[B61] Monner D. Vatz K. Morini G. Hwang S. DeKeyser R. (2013). A neural network model of the effects of entrenchment and memory development on grammatical gender learning. *Biling. Lang. Cogn.* 16 246 10.1017/S1366728912000454

[B62] Munakata Y. Pfaffly J. (2004). Hebbian learning and development. *Dev. Sci.* 7 141–148 10.1111/j.1467-7687.2004.00331.x 15320372

[B63] Neely J. H. Durgunoglu A. (1985). Dissociative episodic and semantic priming effects in episodic recognition and lexical decision tasks. *J. Mem. Lang.* 24 466–489 10.1016/0749-596X(85)90040-3

[B64] Pavlenko A. (2009). “Conceptual representation in the bilingual lexicon and second language vocabulary learning,” in *The Bilingual Mental Lexicon: Interdisciplinary Approaches* ed. Pavlenko A. (Tonawanda, NY: Multilingual Matters) 125–160

[B65] Reznick J. S. Goldfield B. A. (1992). Rapid change in lexical development in comprehension and production. *Dev. Psychol.* 28 406–413 10.1037/0012-1649.28.3.406

[B66] Richardson F. M. Thomas M. S. (2008). Critical periods and catastrophic interference effects in the development of self-organizing feature maps. *Dev. Sci.* 11 371–389 10.1111/j.1467-7687.2008.00682.x 18466371

[B67] Ritter H. Kohonen T. (1989). Self-organizing semantic maps. *Biol. Cybern.* 61 241–254 10.1007/BF00203171

[B68] Rumelhart D McClelland J. (1986). “On learning the past tenses of english verbs,” in *Parallel Distributed Processing: Explorations, in the Microstructure of Cognition,* Vol. 2 *in Psychological and Biological Models* eds McClelland L. J. Rumelhart D. E. PDP Research Group (Cambridge: MIT Press) 216–271

[B69] Saxton M. (2010). *Child Language: Acquisition and Development*. London: SAGE Publications

[B70] Shirai Y. (1991). *Primacy of Aspect in Language Acquisition: Simplified Input and Prototype*. Ph.D. dissertation, Applied Linguistics University of California at Los Angeles

[B71] Silberman Y. Bentin S. Miikkulainen R. (2007). Semantic boost on episodic associations: an empirically-based computational model. *Cogn. Sci.* 31 645–671 10.1080/15326900701399921 21635311

[B72] Spitzer M. (1999). *The Mind within the Net: Models of Learning, Thinking, and Acting*. Cambridge, MA: MIT Press PMC1117340 10617552

[B73] Sporns O. (2010). *Networks of the Brain*. Cambridge: The MIT Press

[B74] Thomas M. S. C. Johnson M. H. (2008). New advances in understanding sensitive periods in brain development. *Curr. Dir. Psychol. Sci.* 17 1–5 10.1111/j.1467-8721.2008.00537.x

[B75] Tiňo P. Farkaš I. van Mourik J. (2006). Dynamics and topographic organization of recursive self-organizing maps. *Neural Comput.* 18 2529–2567 10.1162/neco.2006.18.10.2529 16907636

[B76] Wang X. Forster K. I. (2010). Masked translation priming with semantic categorization: testing the sense model. *Biling. Lang. Cogn.* 13 327–340 10.1017/S1366728909990502

[B77] Westermann G. Ruh N. Plunkett K. (2009). Connectionist approaches to language learning. *Linguistics* 47 413–452 10.1515/LING.2009.015

[B78] Xing H. Shu H. Li P. (2004). The acquisition of Chinese characters: corpus analyses and connectionist simulations. *J. Cogn. Sci.* 5 1–49

[B79] Zhao X. Li P. (2009). Acquisition of aspect in self-organizing connectionist models. *Linguist. Interdiscip. J. Lang. Sci.* 47 1075–1112 10.1515/LING.2009.038

[B80] Zhao X. Li P. (2010). Bilingual lexical interactions in an unsupervised neural network model. *Int. J. Biling. Educ. Biling.* 13 505–524 10.1080/13670050.2010.488284

[B81] Zhao X. Li P. (2013). Simulating cross-language priming with a dynamic computational model of the lexicon. *Biling. Lang. Cogn.* 16 288–303 10.1017/S1366728912000624

[B82] Zhao X. Li P. Kohonen T. (2011). Contextual self-organizing map: software for constructing semantic representation. *Behav. Res. Methods* 43 77–88 10.3758/s13428-010-0042-z 21287105

